# Significant improvement of procedural safety in stenting for basilar stenosis: A historically controlled study

**DOI:** 10.1097/MD.0000000000032186

**Published:** 2022-12-16

**Authors:** Kai Wang, Xiao-tong Xu, Min Jin, Ao-fei Liu, Bao-shi Guo, Ming-yue Qu, Feng Gao, Li Xiang, Yun-e Liu, Feng-yuan Man, Wei-jian Jiang

**Affiliations:** a New Era Stroke Care and Research Institute, The PLA Rocket Force Characteristic Medical Center, Beijing, China; b Research Department, The PLA Rocket Force Characteristic Medical Center, Beijing, China; c Department of Interventional Neuroradiology, Beijing Tiantan Hospital, Capital Medical University, Beijing, China.

**Keywords:** basilar stenosis, procedural safety, stenting, stroke

## Abstract

The basilar artery has the most perioperative complications in stenting compared to the other intracranial arteries. We aim to study whether the procedural safety in stenting for basilar stenosis has improved. This study was a single-arm, non-randomized trial that included historically controlled patients for comparison. Between January 2012 and March 2019, 147 consecutive patients with symptomatic basilar stenoses receiving elective stenting treatment were included in current basilar artery stenting (BAS) group. The prospectively collected and registered 120 patients by the same interventional team from September 2001 to November 2011 were set as historical BAS group for control. A total of 267 individuals were included in this study, with a mean age of 59.5 ± 8.1 years. The proportion of patients with lesion length >15 mm was 26.5% (39/147) in the current BAS group versus 4.2% (5/120) in the historical BAS group. We found significant differences between these 2 groups in Mori A (17.7% vs 42.5%) and Mori C patients (42.9% vs 13.3%). The proportion of patients receiving preoperative high-resolution magnetic resonance (HRMRI) evaluation was 83.0% (122/147) in the current BAS group versus 20.8% (25/120) in the historical group (*P* < .05). Balloon-expendable stent (BES) (n = 1), Wingspan (n = 34), and Enterprise (n = 112) stents were placed in the current BAS group. In contrast, only balloon-expendable stent (BES) (n = 48) and Wingspan (n = 72) were deployed in the historical BAS group. The incidence of the safety endpoint (SE) was 4.1% (involving 6 patients) in the current BAS group versus 11.7% (involving 14 patients) in the historical BAS group (*P* < .05). In multivariate analysis, no risk factor was associated with the occurrence of the safety endpoint (SE). When BAS cases operated by the surgical team accumulated to 120 to 150, the incidence of complications decreased significantly. This is the largest sample size study to discuss the safety of BAS. The significantly decreased incidence of complications indicates that the improving technical measures and the accumulation of operation experience are necessary.

## 1. Introduction

Despite receiving medical therapy, patients with symptomatic basilar artery stenosis (SBAS) have a poor prognosis, with a 33% risk of mortality or recurrent stroke in the first year.^[1,2]^ Over the historical 2 decades, basilar artery stenting (BAS) has become a treatment option for patient refractory to medical therapy in some centers. However, a significantly higher and unacceptable risk of periprocedural stroke or death from symptomatic basilar artery stenosis (SBAS) stenting was noted compared to stenting of other major intracranial artery stenoses. In the Stenting versus Aggressive Medical Management for Preventing Recurrent Stroke in Intracranial Stenosis (SAMMPRIS) trial,^[3,4]^ the risk is 20.8% (10/48) in basilar artery versus 6.7% (11/165, *P* = .01) in others. In another large case series,^[5]^ the risk is 13.0% (9/69) in BAS group versus 2.9% (2/70, *P* = .026) in intracranial vertebral artery group. Therefore, it is necessary to improve the safety of BAS. In this study, we investigate the current safety outcome of BAS compared with historical results and revealed the potential risk factors.

## 2. Materials and Methods

### 2.1. Overall design

This study was a single-arm, non-randomized trial that included historically controlled patients for comparison. The study was approved by the Institutional Ethics Committee (KY2013031). All patients involved have signed the informed consent, and their medical information were prospectively collected, including demographics, atherosclerotic risk factors, clinical data, imaging data, and follow-up data. According to the stenting timepoint, the patients who underwent BAS in the study were extracted and divided into the historical BAS group and the current BAS group. The dividing point of the 2 stages was set as January 2012, because the closed-cell designed enterprise stent was introduced. Moreover, there are obvious distinctions between the 2 stages regarding patient inclusion, perioperative management, and surgical concept (details below). The preoperative, intra-operative, and postoperative information were retrospectively analyzed and compared between groups. All reported endpoints were evaluated and validated by a central review committee composed of designated neuroscientists, neurosurgeons, and radiologists unaware of the choice.

### 2.2. Participants

Patients aged from 18 to 85 years old and with the following characteristics were eligible to participate in the study:

(1) An ischemic stroke (IS) or transient ischemic attack (TIA) within 6 months was a qualifying event attributable to >70% of basilar artery stenosis confirmed by angiography.
(2) The interval from qualifying event to stenting was no less than 7 days for mild stroke (NIH Stroke Scale score <9) or 6 weeks for severe stroke (NIH Stroke Scale score >9).
(3) Time to peak (TTP) and mean transit time (MTT) prolongation in perfusion imaging compared with anterior circulation,
(4) At least 1 atherosclerotic risk factor includes hypertension, diabetes, hyperlipidemia, and cigarette smoking.

Patients with the following conditions were excluded:

(1) Urgent stenting due to acute IS or restenosis due to restenosis.
(2) Balloon angioplasty alone without stenting.
(3) The stenosis site was complicated by aneurysm or received other additional treatments.
(4) There was no risk factor for arteriosclerosis.

### 2.3. Preoperative evaluation and perioperative management

In the current BAS group, all patients underwent preoperative high-resolution magnetic resonance (HRMRI) evaluation to obtain detailed information on the vascular cavity, vascular wall, plaque location, and perforator distribution, except those who had magnetic resonance imaging contraindication. To evaluate the clinical value of intracranial atherosclerotic stenosis (ICAS) stenting, we originally established a new pathophysiology classification of ICAS for the purpose of stenting (Tables 1 and 2). The pathophysiology of ICAS was classified as insufficient perfusion, thromboembolism, perforator involvement and mixed type. The screening process of patients suitable for stenting was based on indications, contraindications, risks of the operation, and pathogenic mechanism. As triple antiplatelet therapy (TAT) under thromboelastogram guidance was proved to reduce thromboembolic events without a higher risk of bleeding in patients undergoing stenting, we established the novel antiplatelet technology and widely applied after January 2013.^[6]^ If the inhibition ratio of either arachidonic acid or adenosine diphosphate (ADP) was <50%, indicating a relative resistance to aspirin or clopidogrel, cilostazol (Zhejiang Otsuka Pharmaceutical Co, Ltd, Shanghai, China) 100 mg twice a day was added. Patients were transferred into the neurological intensive care unit (NICU) after the operation. Postoperative blood pressure was controlled to about 80% of preoperative baseline to prevent perfusion pressure breakthrough. Cerebral computed tomography (CT) was performed after the stenting to exclude intracranial hemorrhage, and imaging examination was performed if neurological events attacked.

**Table 1:** Pathophysiology classification of ICAS for the purpose of stenting.

Classification	Clinical manifestation	CT/ MRI	Perfusion imaging	Wall imaging
**Type I:** Hypoperfusion	Stroke or frequent, short- duration TIA (usually lasting for <1 h). Anterior circulation TIA is stereotyped while posterior circulation TIA is not	Watershed infarction or not	Hypoperfusion in the blood supply region of the stenotic artery	Plaque, mostly strengthened
**Type II:** Thromboembolism	Stroke, or infrequent, non-stereotyped, long duration TIA (usually lasting from 1–24 h)	Infarction in the cortex or near the cortex, usually with multiple infarcts spreading in different blood supply regions of arterial branches	No obvious anomalies except infarct regions	Strengthened plaques; sometimes characteristics of thrombus
**Type III:** Deep perforating artery ostia involvement	Lacuna syndrome. Stroke or frequent, stereotyped, short- duration TIA	Infarcts in the blood supply region of the deep subcortical perforating artery, or not	Hypoperfusion in the blood supply region of the deep perforating artery	Plaques are located in the deep perforating artery origin, such as the side or posterior wall of basilar artery, superior wall of middle cerebral artery
Type IV*: Mixed type	Mixed symptoms	Mixed symptoms	Mixed symptoms	Mixed symptoms

* Type IV is divided into 4 categories. Type IV a: I + II; Type IV b: I + III; Type IV c: II + III; Type IV d: I + II + III.

CT = computed tomography, ICAS = intracranial atherosclerotic stenosis, MRI = magnetic resonance imaging, TIA = transient ischemic attack.

**Table 2:** Evaluation of ICAS pathophysiology classification on clinical value of stenting.

Classification	Evaluation on clinical value
**Type I + IVa**	Can both benefit from a successful stenting, because hypoperfusion is an independent predictor of stroke recurrence and a successful stenting will improve perfusion in the affected area
**Type II**	May not benefit from stenting, because active medical treatment can stabilize the atherosclerotic plaques causing the embolization, and on the contrary, stenting procedure may induce embolus shedding from an unstable plaque
**Type III + IVc**	Stenting may cause further damage and aggravate ischemia of the perforating branches’ blood supply region because of its “snow plow effect”
**Type IVb + IVd**	Stenting can improve perfusion in the distal area of stenotic artery, and can carry a higher risk of perforator stroke; the benefit and the risk of ICAS stenting should be weighted

ICAS = intracranial atherosclerotic stenosis.

Correspondingly, in the historical BAS group, preoperative evaluation through HRMRI was only performed for cases after December 2007. All patients received conventional antiplatelet therapy (100 mg aspirin plus 75 mg clopidogrel daily for ≥3 days before operation and for at least 3 months after stenting and then either 100 mg aspirin or 75 mg clopidogrel for life).^[7]^ The new pathophysiology classification of ICAS for the purpose of stenting has not been applied. Other perioperative management strategies, including blood pressure (BP) control and patient monitoring, were similar with the current BAS group. We specially designated personnel to control the quality of perioperative management of patients to ensure the standardization of patient management.

### 2.4. Stenting procedure and devices selection

All stenting procedures were performed by the same neurological interventional team. The operator selected the stent implantation procedure according to the lesion characteristics and the operator’s experience. In the current BAS group, balloon-expendable stent (BES), open-cell self-expanding stent (SES), and closed-cell SES were optional, and closed-cell designed enterprise (introduced in January 2012) was the most selected. Synchronization with enterprise, angiographic 3-dimensional (3D) road mapping was introduced for intraoperative guidance. During procedures, guiding catheter was first advanced to the distal of the vertebral artery V2 segment, and an undersized Gateway balloon (Boston Scientific) at low pressures was used for predilation of the stenotic site before stent deployment. Suboptimal balloon angioplasty referred to the expansion of lesions with a balloon 31 to 50% smaller than the reference vessel diameter, which can effectively prevent the rupture of the target lesion of negative remodeling and the occlusion of the perforating artery. After pre-dilation, stents were usually selected with a diameter of 0.5 to 1.0 mm wider than reference and with 3 mm longer on both sides of lesion length.

In the historical BAS group, BES or open-cell SES was available for stenting, and 2-dimensional (2D) pathway mapping was used for intraoperative guidance. For BES, the stent diameter was the same or slightly smaller than normal adjacent vessels (1:1 or 0.9:1). The stent straddled the stenotic segment and was then deployed by gradual balloon inflation (up to 6–8 atm). In view of the short length of BES in earlier period, another stent was used when the first one could not completely cover the lesion. The regarded terminal point of stenting procedure, of which the ideal is completely coverage of the target lesion with stent, is <50% residual stenosis and good anterograde blood flow.

### 2.5. Follow-up and safety endpoint (SE)

Clinical follow-up information was derived from daily patient exams until discharge and 30-day follow-up visits. Safety endpoint (SE) was defined as any stroke or mortality within 30 days of stenting. The definition of stroke is consistent with previous literature.^[8]^ IS is defined as a new focal neurological deficit of sudden onset, lasting at least 24 hours, unassociated with hemorrhage on computed tomography (CT) or magnetic resonance imaging (MRI). Hemorrhagic stroke is defined as a new brain hemorrhage involving parenchymal hemorrhage, subarachnoid hemorrhage, or intraventricular hemorrhage, which is associated with a seizure or with symptoms or signs lasting 24 hours or longer.^[9]^

### 2.6. Data analysis

In the present study, statistical analyses were performed using IBM SPSS version 18.0 (IBM Corp., Armonk, NY). Characteristics are presented as means, medians or percentages based on types of variables. The level of significance was set at *P* < .05. Pearson χ^2^ test or Fisher exact test was used for categorical variables comparison. After contrastive analyzing between groups, 2 sets of data were pooled. Continuous variables were transferred to dichotomous variables. Fisher exact test was used to evaluate univariate associations between baseline characteristics, perioperative and procedural variables and safety outcome. Multivariate logistic regression analysis was used to assess the relationship between outcome and factors. The multivariate model was constructed with variables with *P*-values <.2 in Fisher exact test using forward inclusion.

To intuitively display the cumulative change of stenting complications in different experience stages, we calculated the incidence of complications according to the cumulative number of every 30 cases, and drew the experience curve of operative safety. The slope of the curve represents the rate of surgical experience accumulation.

## 3. Results

### 3.1. Baseline characteristics

A total of 267 individuals with a mean age of 59.5 ± 8.1 years were included in this study. The stenting was performed at a median of 21 days after the qualifying event. One hundred forty-seven consecutive patients were included in the current BAS group, and 120 patients in the historical BAS group. There were statistical differences between 2 groups in baseline data such as patient age, gender, hyperlipidemia and qualifying event (Table 3).

**Table 3:** Baseline characteristics of patients included.

Variables	Current stage N = 147	Historical stage N = 120	*P*-value	Merge group N = 267
Age, ≥60 yr, n (%)	79 (53.7%)	48 (40.0%)	***P* < .05** *	127 (47.6%)
Female, n (%)	28 (19%)	12 (10.0%)	***P* < .05** *	40 (15.0%)
Hyperlipidemia, n (%)	95 (64.6%)	101 (84.2%)	***P* < .05** *	196 (73.4%)
Hypertension, n (%)	117 (79.6%)	86 (71.7%)	*P* > .05	203 (76.0%)
Diabetes mellitus, n (%)	56 (38.1%)	36 (30.0%)	*P* > .05	92 (34.5%)
Cigarette smoking, n (%)	63 (42.9%)	57 (47.5%)	*P* > .05	120 (44.9%)
Stroke as a qualifying event, n (%)	86 (76.8%)	106 (88.3%)	***P* < .05** *	192 (71.9%)

* statistically significant difference.

### 3.2. Comparison of lesion characteristics

The proportion of patients with lesion length >15 mm was 26.5% (39/147) in the current BAS group versus 4.2% (5/120) in the historical BAS group (*P* < .05). Furthermore, there was no significant difference in the proportion of lesions with stenosis rate >80% (51.7% vs 38.3%). Moreover, the basilar stenosis was classified by Mori type using characteristics and angulation. We found significant differences between the groups in Mori A (17.7% vs 42.5%) and Mori C patients (42.9% vs 13.3%) (Table 4).

**Table 4:** Comparison of stenosis characteristics of patients.

Variables	Current stage N = 147	Historical stage N = 120	*P*-value	Merge group N = 267
Lesion length, ≥15mm, n (%)	39 (26.5%)	5 (4.2%)	***P* < .05** *	44 (16.4%)
Preoperative stenosis per, ≥80%, n (%)	76 (51.7%)	46 (38.3%)	*P* > .05	122 (45.7%)
Mori type				
Mori A	26 (17.7%)	51 (42.5%)	***P* < .05** *	77 (28.9%)
Mori B	58 (39.5%)	53 (44.2%)	*P* > .05	111 (41.6%)
Mori C	63 (42.9%)	16 (13.3%)	***P* < .05** *	79 (29.6%)

* statistically significant difference.

### 3.3. Perioperative strategies and operative decision

Considering technical improvement strategies, the proportion of patients receiving preoperative HRMRI evaluation was 83.0% (122/147) in the current BAS group versus 20.8% (25/120) in the historical group (*P* < .05). And the proportion of patients receiving perioperative antiplatelet therapy under thrombelastogram guidance was 54.4% (80/147) versus 0% (*P* < .05).

Of 147 cases in the current BAS group, BES (n = 1), Wingspan (n = 34) and Enterprise (n = 112) stent were deployed. In contrast, only BES (n = 48) and Wingspan (n = 72) were deployed in the historical BAS group. There were significant differences in stent selection between the 2 groups (*P* < .05). Moreover, according to the angiographic result after stenting, the proportion of lesions with postoperative residual stenosis >30% was 62.6% (92/147) in the current group versus 29.2% (35/120) in the historical group (*P* < .05) (Table 5).

**Table 5:** Comparison of perioperative strategies and operative decision.

Variables	Current stage N = 147	Historical stage N = 120	*P*-value	Merge group N = 267
**Perioperative strategies**				
Preoperative HRMRI evaluation, n (%)	122 (83.0%)	25 (20.8%)	***P* < .05** *	147 (55.1%)
AT under the guidance of TEG, n (%)	80 (54.4%)	0 (0%)	***P* < .05** *	80 (30.0%)
**Procedural strategies**				
Stent type				
BES	1 (0.7%)	48 (40.0%)	***P* < .05** *	49 (18.4%)
Wingspan	34 (23.1%)	72 (60.0%)	***P* < .05** *	106 (39.7%)
Enterprise	112 (76.2%)	0 (0%)	***P* < .05** *	112 (41.9%)
Residual stenosis per, ≥30%, n (%)	92 (62.6%)	35 (29.2%)	***P* < .05** *	127 (47.6%)

AT = antiplatelet therapy, BES = balloon-expendable stent, HRMRI = high-resolution magnetic resonance, TEG = thrombelastogram.

* statistically significant difference.

### 3.4. SE

The total incidence of SE was 7.5% (20/267). Of all 20 SEs, there were 11 perforator IS, 6 non-perforator IS (including 1 IS-related death), and 3 intracerebral hemorrhage (ICH) (including 2 ICH-related deaths) (Table 6). In multivariate regression analysis, no risk factors were associated with the SE among the baseline and treatment variables.

**Table 6:** Comparison of safety endpoint.

Variables	Current stage N = 147	Historical stage N = 120	*P*-value	Merge group N = 267
**Safety endpoint, n (%**)	**6 (4.1%**)	**14 (11.7%**)	***P* < .05** *	**20 (7.5%**)
Perforator IS	3	8		11
Non-perforator IS	2	4 (1 death)		6
ICH	1 (1 death)	2 (1 death)		3

ICH = intracerebral hemorrhage, IS = ischemic stroke.

* statistically significant difference.

The incidence of the SE was 4.1% (involving 6 patients) in the current BAS group versus 11.7% (involving 14 patients) in the historical BAS group (*P* < .05). Six SEs occurred in the current group included 3 perforator IS, 2 non-perforator IS, and 1 ICH. Comparatively, there were 8 perforator IS, 4 non-perforator IS, and 2 ICH in the historical group.

**Figure 1.**
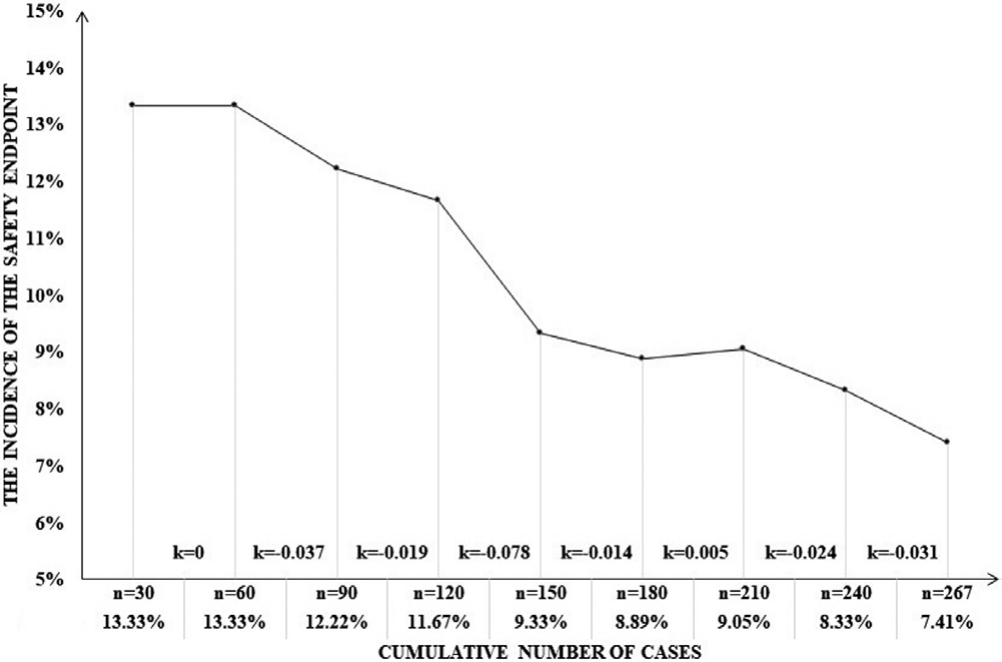
The incidence of complications according to the cumulative number of every 30 cases.

As shown in experience curve of operative safety (Fig. 1), the incidence of SE decreased from 13.3% of the initial 30 cases to 7.5% when cumulated to 267 patients. As cases accumulated, the curve generally showed an apparently downward trend. Of note, when cases of BAS accumulate to 120 to 150, the incidence of complications decreased significantly (slope maximum).

## 4. Discussion

A significant improvement in stenting procedural safety for basilar stenosis is confirmed in this study. We observed that the incidence of perioperative complications of BAS decreased significantly from 11.4% to 4.1%. The trend in our study is consistent with other high-quality studies. A systematic review published in 2009,^[10]^ including the lower-risk vertebral artery subgroup, summarized a median of 8.3% periprocedural complication risk for posterior circulation. Correspondingly, considering perforator stroke, the most common complication associated with basilar stenting, researchers from high flow center identified 5.1% of perioperative perforator stroke incidence (255 patients included).^[11]^ Even more impressively, several recent studies for intracranial stenting have reported a lower risk of periprocedural complications at 2% to 4.3%.^[8,12,13]^ These results imply that the lesion vessel may no longer be a major concern about stenting decision-making.

A series of improvements in technical measures may have contributed to the favorable short-term results of basilar stenting, which include deepening disease understanding, optimized operative skill, advanced preoperative evaluation technique, progressive stent system, etc. Groschel et al point out that a mechanism of perforator stroke complication refers to plaque which involved in perforator ostia of basilar artery dorsal or lateral walls.^[11]^ In addition, the HRMRI and 3-dimensional road-mapping technology have contributed significantly to precise preoperative evaluation.^[14–16]^ Moreover, the established pathophysiology classification of ICAS for the purpose of stenting provides detailed evaluation references. Finally, individualized operative techniques, including applying undersized angioplasty balloons and slow balloon inflation technique (especially dorsal or lateral lesion), help to minimize complication risk, especially in complex lesions.^[17,18]^

None of the baseline characteristics or treatment strategies was identified to be associated with the SE herein. However, the Stenting versus Aggressive Medical Management for Preventing Recurrent Stroke in Intracranial Stenosis (SAMMPRIS) study implied that never smoking, basilar stenosis, diabetes, and older age were associated with periprocedural ischemic events.^[4,19]^ Another retrospective study found preexisting diabetes and basilar artery lesion site were risk factors associated with postprocedural complications after stenting in severe symptomatic intracranial stenosis.^[19]^ On the one hand, that may be related to our study only included patients of the basilar stenting subgroup. On the other hand, several technical improvement measures in this study were homogeneous variables (synchronous improvement over time), which may interfere with each other. Additionally, given that operator’s level of experience significantly affected the outcome, it was unconvincing when not evaluated and included in the analysis. Under the current conditions, we should not expect to use a single indicator to determine the safety outcome; therefore, we need to focus on the whole process of technical improvement, from preoperative evaluation to postoperative management.^[20]^

As is known, a certain number of case accumulation is necessary to improve the safety of high-risk exploratory interventional operations. In the present study, we found the complication curve generally showed an apparently downward trend and when cases accumulated to 120 to 150, the incidence of complication decreased significantly. The results are instructively important for developing training programs of other operation teams. Of note, the present complication curve differs from the previously defined learning curve. Cai study suggested learning curve of ICAS to overcome complications in a risk-adjusted manner is 21 cases, which means experience transfer on behalf of mature technology.^[21]^ Instead, the complication curve here means that from the very beginning with few experiences for references, as one of the earliest teams in China to carry out BAS, it is a long course of achieving acceptable safety outcomes.

This study was limited by a single-center design, possibly incorporating inclusion bias. We only examined the clinical outcome within 30 days; therefore, the long-term risk of complications after basilar stenting remained unreported in the present study. In addition, the operator’s experience, which might be the most crucial connotation of quality improvement, could not be quantified and brought into variate analysis. For other improved strategies (HRMRI, the new pathophysiology classification, etc.), the detailed information could not be reviewed individually and thus their actual effect value cannot be assessed quantitatively through the present study.

## 5. Conclusion

This study is the largest sample size to study procedure safety of basilar stenting. The significantly decreased incidence of complications was confirmed. Solving the safety problem of high-risk interventional operation, the improving technical measures and the accumulation of operation experience are necessary.
